# Effects of 5-Azacytidine on Growth and Hypocrellin Production of *Shiraia bambusicola*

**DOI:** 10.3389/fmicb.2018.02508

**Published:** 2018-10-18

**Authors:** Yan Jun Ma, Can Song Lu, Jian Wen Wang

**Affiliations:** College of Pharmaceutical Sciences, Soochow University, Suzhou, China

**Keywords:** *Shiraia bambusicola*, DNA methylation, 5-azacytidine, fluffy, hypocrellin, reactive oxygen species, transcriptome analysis

## Abstract

Hypocrellins, fungal perylenequinones of *Shiraia bambusicola* are developed as important photodynamic therapy agents against cancers and viruses. Due to the limitation of the wild resources, the mycelium culture is a promising alternative for hypocrellin production. As DNA methylation has profound effects on fungal growth, development and secondary metabolism, we used both McrBC cleavage and HPLC analysis to reveal the status of DNA methylation of *S. bambusicola* mycelium. We found that DNA methylation is absent in mycelia, but DNA methylation inhibitor 5-azacytidine (5-AC) still induced the fluffy phenotype and decreased hypocrellin contents significantly. Simultaneously, a total of 4,046 differentially expressed genes were induced by 5-AC, including up-regulated 2,392 unigenes (59.12%) and down-regulated 1,654 unigenes (40.88%). Gene ontology analysis showed 5-AC treatment changed expression of genes involved in membrane composition and oxidation–reduction process. The fluffy phenotype in 5-AC-treated *S. bambusicola* was closely related to strong promotion of developmental regulator *WetA* and the repression of the sexual developmental actor *VeA* and *LaeA*. It was a surprise finding that 5-AC reduced reactive oxygen species (ROS) production significantly in the mycelia via the inhibition of NADPH oxidase gene (*NOX*) expression and NOX activity. With the treatment of vitamin C and H_2_O_2_, we found that the reduced ROS generation was involved in the down-regulated expression of key genes for hypocrellin biosynthesis and the decreased hypocrellin production. To our knowledge, this is the first attempt to examine DNA methylation level in *S. bambusicola*. Our results suggested that the mediation of ROS generation could not be ignored in the study using 5-AC as a specific DNA methylation inhibitor.

## Introduction

Hypocrellins are a series of natural perylenequinone pigments including hypocrellin A (HA), B, and C isolated from the fruiting body of *Shiraia bambusicola*, a pathogenic fungus of bamboo. The fruiting body has been used in traditional Chinese medicine for treating rheumatic pain, stomachache, vitiligo, and psoriasis (Zhen and Di, 1995). Hypocrellins have attracted intense interest as clinically useful photosensitizers in photodynamic therapy (PDT) on different skin diseases (Wan and Chen, 1981; Wang and Zhang, 1992). Furthermore, as a new type of non-porphyrin photosensitizers, hypocrellins have several advantages with higher yield of singlet-oxygen and triplet quantum, higher phototoxicity but lower dark toxicity (Di et al., 1990; Miller et al., 1995). Therefore, hypocrellin biosynthesis has received intense attention recently (Yang et al., 2014; Zhao et al., 2016).

Epigenetic modifications including DNA methylation, histone modifications, and nucleosome positioning have profound effects on fungal growth, development, and secondary metabolism (González-Menéndez et al., 2016). 5-azacytidine (5-AC), a derivate of nucleoside cytidine is an inhibitor of DNA methyltransferase, which is an important chemotherapeutic drug and widely used to explore the role of DNA methylation (Christman, 2002). Kritskii et al. (2001) reported that 5-AC inhibited significantly the growth of *Neurospora crassa* but promoted spore germination at 3–300 μM. The “fluffy” phenotype of *Aspergillus fumigatus* was induced by 5-AC at the concentrations up to 500 μM (Ben-Ami et al., 2010). The carotenoid production in *N. crassa* could be stimulated by 5-AC at concentrations lower than 30 μM, but suppressed at 100 and 300 μM, respectively (Kritskii et al., 2001). The production of aflatoxins by *A. flavus* was inhibited after 5-AC treatment at 1 mM (Lin et al., 2013a). However, there has been so far neither report on the epigenetic modifications of *S. bambusicola*, nor regarding the regulation of DNA methylation on the growth and hypocrellin production of *S. bambusicola*. Therefore, as a follow-up to our efforts on exploring the biosynthesis of hypocrellin (Lei et al., 2017; Sun et al., 2017), we wish to analyze the status of DNA methylation in *S. bambusicola* and its regulation on fungal growth and hypocrellin production. The possible physiological responses induced by 5-AC are also investigated in detail.

## Materials and Methods

### Fungal Strain, Media, and Culture Conditions

The hypocrellin-producing strain *S. bambusicola* S8 was isolated from the shoot tissues of bamboo (*Brachystachyum densiflorum*) (Sun et al., 2017), which was stocked in China General Microbiological Culture Collection Center (CGMCC3984, Beijing, China). The strain was initially grown on potato dextrose agar (PDA) medium in a Petri dish at 28°C for 8 days. A small piece (5 mm × 5 mm) of the strain from the PDA plate was dug and transferred into a 150-mL Erlenmeyer flask containing 50 mL potato dextrose broth (Yang et al., 2013). The liquid culture was incubated for 8–10 days at 28°C in a rotary shaker at 150 rpm.

### McrBC Analysis of DNA Methylation

Total DNA was extracted from the mycelia on day 8 using Plant Genomic DNA Kit (Tiangen, Beijing, China) according to the manufacturer’s protocol. DNA integrity and purity were confirmed using the Agilent 2100 Bioanalyzer (Agilent Technologies, Santa Clara, CA, United States). The enzyme digestion reaction system was established based on the protocol of McrBC (New England Biolabs, Beijing, China). The reaction was performed in a final volume of 20 μl, comprising 500 ng of hyphal DNA, 2 μL of 10 × New England Biolabs (NEB) buffer 2, 2 μL of 10 × GTP (10 mM), 2 μL of 10 × bovine serum albumin (BSA, 1 mg/mL) and 2 μL of McrBC (10 U/μL), made up to the final volume with double distilled water (Manoochehri et al., 2013). The reaction liquid was kept at 37°C for 2 h and was then electrophoresed on a 0.75% agarose gel for 40 min at 80 V.

### HPLC Analysis of DNA Methylation

The extent of methylation in DNAs was determined by HPLC method (Kuo et al., 1980) and performed with the reverse-phase HPLC system (Agilent1260, Wilmington, MA, United States) with UV detection at 280 nm, using the Agilent HC-C18 column (250 mm × 4.6 mm dimension) at a flow rate of 1 mL/min with a mobile phase of 50 mM KH_2_PO_4_: methanol at 90: 10 (v/v). DNA methylation was quantified with the nucleoside standards (2′-deoxyadenosine, 5′-methyl-2′-deoxycytidine, 2′-deoxycytidine, 2′-deoxyguanosine, and 2′-deoxythymidine) purchased from Sigma (St. Louis, MO, United States).

### 5-AC Treatment

5-AC (purity, more than 98%, St. Louis, MO, United States) was dissolved in dimethyl sulfoxide (DMSO) at 200 mM as stock solution. To measure the effects of 5-AC on fungal biomass and hypocrellin production, 5-AC solution was added to the cultures on day 3 post-inoculation to make up the desired concentration. The same volume of DMSO (0.5%, v/v) was added to control group. All experiments were carried out in shake-flask cultures on a rotary shaker at 150 rpm and at 28°C for 8 days. Each experiment was carried out in triplicate and all results were expressed as mean ± standard deviation (SD).

### Measurement of Fungal Biomass, Medium pH, and Residual Glucose

The cultural medium was harvested by filtration with 400-mesh filter membrane (Dongkang, Tianjin, China) for the assay. The mycelia were dried to constant weight in 60°C oven to evaluate the fungal biomass [dry weight (DW)]. The medium pH was detected with pH electrode meter (FE20, Metteler Toledo, Zürich). The residual glucose content was determined by the anthrone-sulfuric acid method (Ebell, 1969).

### Observation on Fungal Morphology

The fungal spore suspension (200 μL of 1.5 × 10^3^ spores/mL) was spread on the PDA plates with or without 0.8 mM 5-AC. After the inoculation, the plates were cultivated in the dark at 28°C for 10 days. The morphological characteristic of *S. bambusicola* S8 was observed and photographed using stereoscopic microscope (SMZ1000, Nikon, Tokyo, Japan) and light microscope (CKX41, Olympus, Tokyo, Japan).

### Measurement of ROS Generation and Activities of Redox-Related Enzymes

The generation of reactive oxygen species (ROS) in mycelial cells was detected by 2, 7-dichlorodihydrofluorescein diacetate (DCFH-DA, Beyotime Biotechnology, Haimen, China) (You et al., 2013). 5-AC at 0.8 mM was added on day 3 of the mycelium cultures. After 2 days of 5-AC treatment, the mycelia were incubated with DCFH-DA at 10 μM for 1 h. The fluorescence in mycelia was observed using a fluorescent microscope (CKX41, Olympus, Tokyo, Japan) with excitation wavelength at 485 nm and emission wavelength at 528 nm.

In order to measure the accumulation of ROS of mycelia with 5-AC treatment, the fresh mycelia (0.5 g) were ground with 0.05 M phosphate buffer (PBS, pH 7.8) and resulting crude homogenate was centrifuged at 12,000 ×*g* for 20 min at 4°C. The supernatant was used for the assay. H_2_O_2_ content was determined as previously described method (Pan et al., 2014). The content of superoxide anion (O_2_^-^) was determined using hydroxylamine reaction method (Wu and Tiedemann, 2002). The activities of the redox-related enzymes such as NADPH oxidase (NOX), superoxide dismutase (SOD), catalase (CAT), and glutathione peroxidase (GSH-Px) were measured according to the previously described methods, respectively (Paglia and Valentine, 1967; Marklund and Marklund, 1974; Aebi, 1984; Duan et al., 2009).

### Detection of Hypocrellin Contents

The extraction and analysis of hypocrellins in intracellular (mycelium) and extracellular (cultural broth) were based on the method described in our previous report (Sun et al., 2017). Briefly, HA in mycelium was extracted from harvested mycelia with ethanol and HA in the medium was extracted by methylene chloride at the same time. After the extracting solvent was removed using evaporation under vacuum, the remaining solid extract was stored in tight vials at -20°C. The sample was redissolved in methanol for hypocrellin quantification by reverse-phase HPLC (Agilent 1260, Wilmington, MA, United States) quipped with 250 mm × 4.6 mm Agilent HC-C18 column. Samples were eluted with a mobile phase of CH_3_OH: water at 75:25 (v/v) and monitored at 465 nm. Hypocrellins were quantified with genuine standards provided by the Chinese National Compound Library (Shanghai, China).

### Transcriptome Sequencing and Analysis

The samples were collected from 4-day-old mycelia cultivated with or without 5-AC treatment. Three independent experiments on control or 5-AC-treated sample were conducted to establish cDNA libraries. The libraries were sequenced using HiSeq X Ten platform (Illumina, San Diego, CA, United States). Raw reads were first cleaned by removing adaptor fragments to obtain high-quality clean reads, reads containing more than 5% ambiguous base and law-quality reads containing more than 20% bases with a *Q* value ≤ 10. *De novo* assemble was performed using the Trinity program (version: trinityrnseq_r20131110) (Grabherr et al., 2011). Unigenes were further processed to form longer sequences by software TGICL (Pertea et al., 2003). All unigenes were assigned to putative gene description following BLASTX alignment to the Non-redundant (NR^1^), Swiss-Prot^2^, Cluster of Orthologous Groups of Proteins (KOG^3^), Kyoto Encyclopedia of Genes and Genomes (KEGG^4^) and Gene Ontology (GO^5^) databases with a cut off *E* value of ≤ 1*e*^-5^. The raw RNA-seq data were submitted to NCBI’s Gene Expression Omnibus (GEO) repository under accession number SRP 151186^6^. Gene expression levels were calculated through the fragments per kilobase per million reads (FPKM) method to normalize the read counts between the samples (Mortazavi et al., 2008). In this work, the significance of gene expression differences was assessed using the | foldchange|≥ 2 and *p*-value < 0.05.

### Quantitative Real-Time PCR

Total RNA was extracted from the fungal mycelia using RNAprep pure Plant Kit (Tiangen, Beijing, China) according to the manufacturer’s protocol and the cDNA was synthesized using the reverse transcriptase (Fermentas, Burlington, ON, Canada). Specific primers were designed with the Primer Express software (Applied Biosystems, Foster City, CA, United States) and the primer sequences were listed on **Supplementary Table S1**. The qRT-PCR condition was set based on the protocol of FastStart Universal SYBR Green Master (Roche, Basel, Switzerland) and the amplifications were performed in CFX96 Touch Real-Time PCR Detection System (Bio-Rad, Hercules, CA, United States). All reactions were performed under the following conditions: 3 min at 95°C, followed by 40 cycles of 30 s at 95°C, 30 s at 56°C and 15 s at 72°C. The relative gene expressions were calculated from cycle threshold values using the 2^-ΔΔCT^ method (Zhang et al., 2016).

### Statistical Analysis

Each experiment was carried out in triplicate and all results were expressed as mean ± standard deviation (SD). One-way ANOVA followed by *t*-test, *p* < 0.05 being considered statistically significant.

## Results

### Effects of 5-AC on Fungal Growth and Hypocrellin Production

**FIGURE 1:**
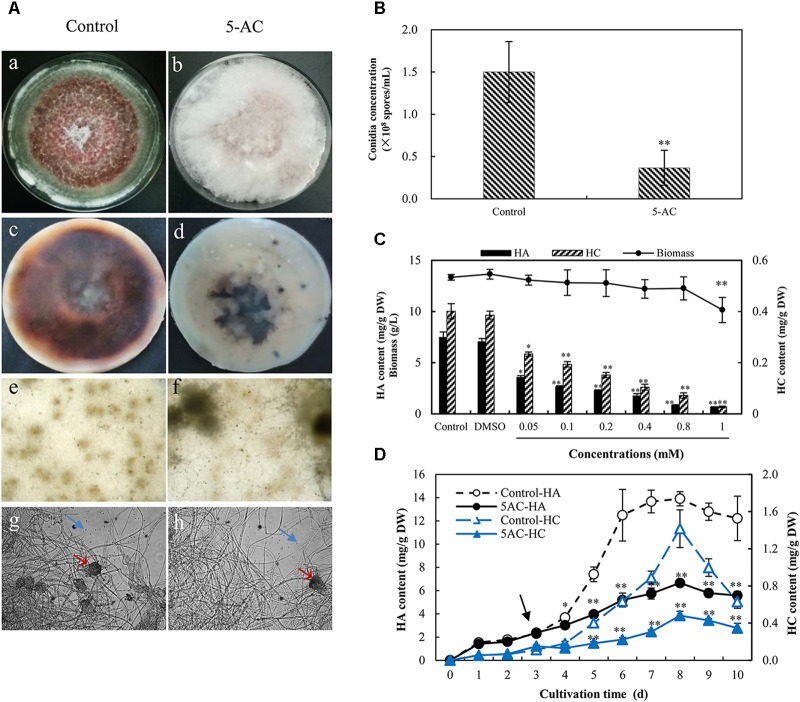
Effects of 5-AC on mycelial growth and hypocrellin contents of *Shiraia bambusicola* S8. **(A)** Morphologic characteristics and red pigments secretion. The mycelia of *S. bambusicola* were kept on PDA with or without 5-AC (0.8 mM) for 10 days. (a,b) Macroscopic colony appearance; (c,d) the secretion of pigments on PDA medium; (e,f) hyphal morphology on dissecting microscopy (40×); (g,h) the pycnidium formation and conidium generation. *Arrow* (red) indicates the pycnidium. *Arrow* (blue) indicates the conidium. **(B)** Effect of 5-AC (0.8 mM) on the generation of conidia of *S. bambusicola* on day 8. **(C)** Effects of 5-AC at different concentrations (0–1.0 mM) on mycelia biomass, hypocrellin contents. **(D)** Time profiles of mycelia biomass and hypocrellin contents in *S. bambusicola* submerged cultures with the addition of 5-AC at 0.8 mM. 5-AC was added on day 3 of the culture. *Arrow* (black) indicates the time point of 5-AC addition. Values are mean ± SD from three independent experiments. ^∗^*p* < 0.05, ^∗∗^*p* < 0.01.

The morphology characteristics of *S. bambusicola* S8 was observed on PDA plates (**Figure 1**). After the incubation without 5-AC for 10 days, *S. bambusicola* presented abundant red substrate mycelia and poor aerial mycelia (**Figure 1A-a**). However, 5-AC at 0.8 mM induced the strain to the white “fluffy” phenotype lacking red substrate hyphae (**Figure 1A-b**). The secretion of red pigments on PDA plate (**Figure 1A-c**) was inhibited seriously by 5-AC (**Figure 1A-d**). After 5-AC treatment the mycelia grew in a cluster rather than in a comparatively homogeneous way in the control (**Figure 1A-e,f**). Moreover, compared to the control group (**Figure 1A-g**), the white “fluffy” phenotype was characterized by lacking of pycnidium formation and conidium germination (**Figure 1A-h**). The concentration of conidia on solid media decreased rapidly from 1.5 to 0.4 × 10^8^ spores/mL after 5-AC treatment (**Figure 1B**).

As shown in **Supplementary Figure S1**, *S. bambusicola* S8 has a typical time courses of mycelia growth and hypocrellin production in submerged cultures. HA and HC contents increased to the highest value of 13.97 and 1.30 mg/g DW respectively with time up to day 8, and then decreased appreciably. HB was not detected from this strain and no release of hypocrellins was detected in PDB medium during the submerged culture. When the cultures were treated with 5-AC at 0.05–1 mM on day 3, the retardation of mycelium growth was observed only at 1 mM (**Figure 1C**). However, hypocrellin contents in mycelia were inhibited significantly at all tested concentrations (0.5–1 mM) of 5-AC. Both HA and HC production were inhibited during all treated period (**Figure 1D**). Simultaneously, we could not find any changes of the medium pH and glucose consumption by 5-AC (**Supplementary Figures S2**, **S3**).

### DNA Methylation of *S. bambusicola* in Mycelium Cultures

**FIGURE 2:**
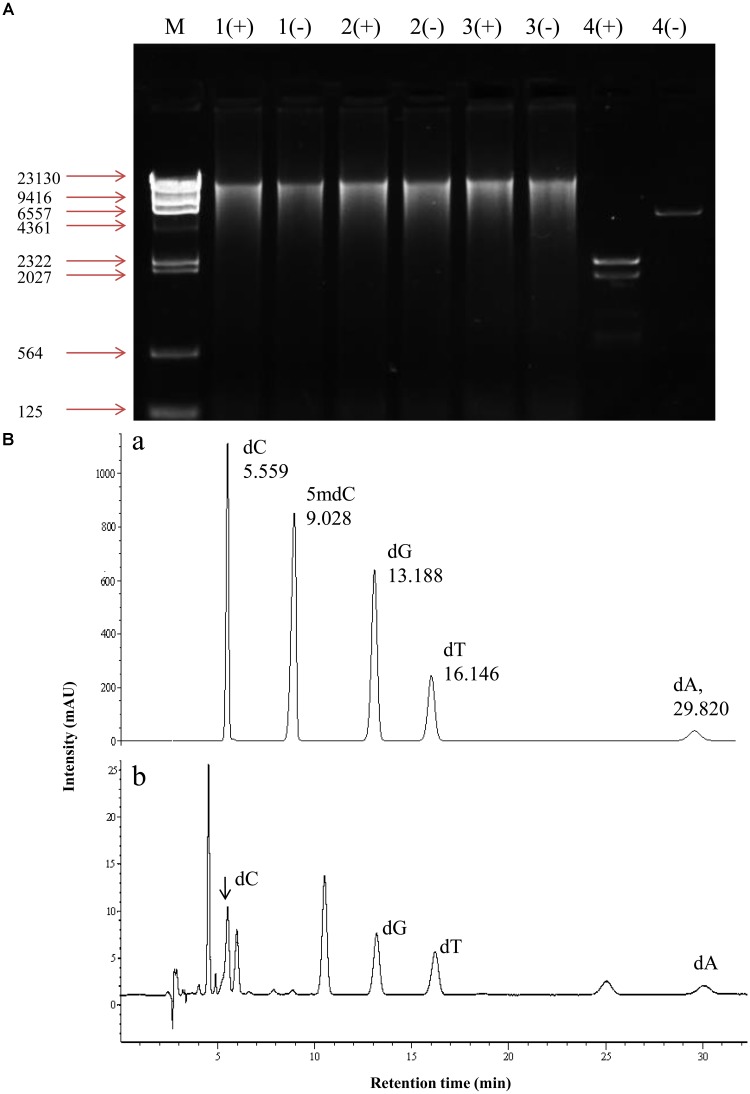
DNA methylation in mycelium of *S. bambusicola* S8 after 5-AC treatment. **(A)** McrBC digestion of genomic DNA of S8 strain. (+), McrBC digestion system; (–), a system contains digestion buffer but not McrBC enzyme. Genomic DNA from mycelia treated with 5-AC (lane 1+, –) or without 5-AC (lane 2+, –). 5-AC at 0.8 mM was added on day 3 and mycelium cultures continued to day 8. DMSO-treated group was used as negative control (lane 3+, –). A plasmid with a digestion site of McrBC was used as positive control (lane 4+, –). **(B)** Detection of 5-mdC in the genomic DNA of S8 strain. (a) Chromatogram of five standard deoxynucleotides. dA, dC, dG, 5-mdC and dT represent 2′-deoxyadenosine, 2′-deoxycytidine, 2′-deoxyguanosine, 5′-methyl-2′-deoxycytidine and 2′-deoxythymidine, respectively. (b) The chromatogram of hydrolysed DNA isolated from *S. bambusicola* S8.

In order to determine the status of DNA methylation of *S. bambusicola*, we performed a preliminary qualitative analysis using McrBC enzyme digestion method (**Figure 2A**). McrBC is an endonuclease to cleave DNA containing methyl-cytosine on one or both strands (Sutherland et al., 1992). Our results showed that mycelial DNA in cultures with or without 5-AC treatment could not be digested enzymatically by McrBC (lane 1, 2 in **Figure 2A**). The linear plasmid with a digestion site of McrBC (positive control) was cut into several fragments between approximately 500 bp and 2.3 kb in size (lane 4 in **Figure 2A**). Furthermore, the content of 5-mdC in mycelia was quantitatively analyzed by HPLC (**Figure 2B**). The HPLC peak eluting of five standard deoxynucleotides at 5.559, 13.188, 16.146, 29.820, and 9.028 min correspond to dC, dG, dT, dA, and 5-mdC, respectively (**Figure 2B-a**). The eluting peak of 5-mdC was almost absent in the genomic DNA of mycelia (**Figure 2B-b**). Our result indicated preliminarily that the level of DNA methylation is very low or even absent in *S. bambusicola* S8 mycelia.

### Transcriptional Changes Induced by 5-AC

**FIGURE 3:**
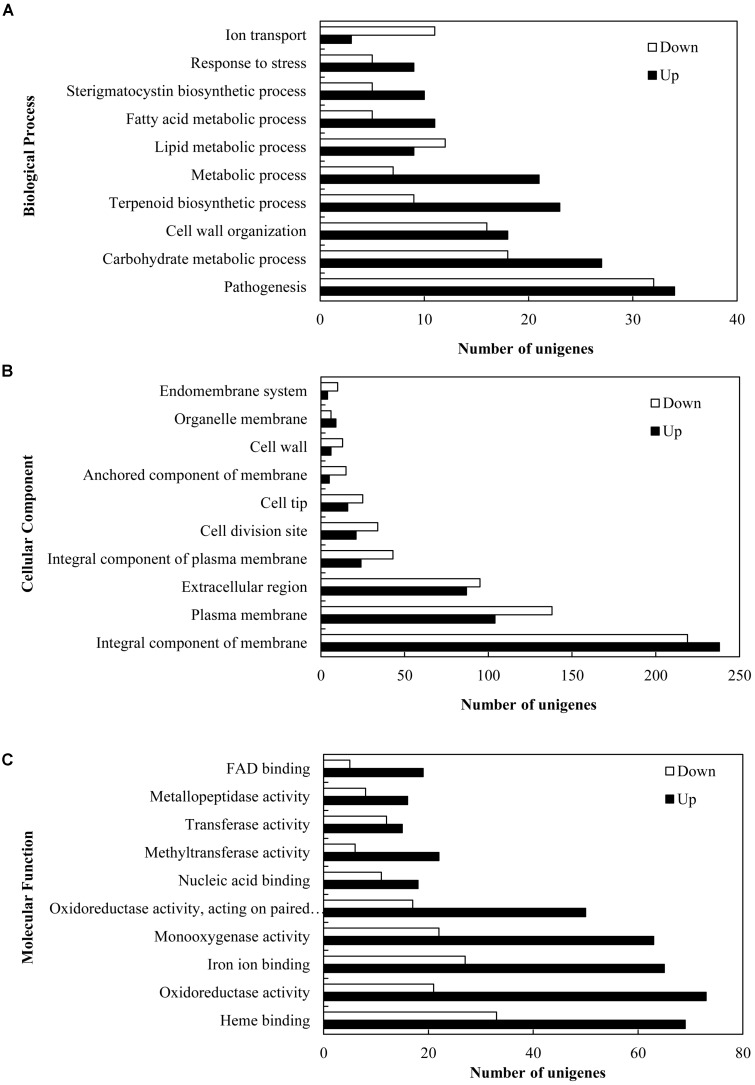
Gene ontology (GO) function categories of the DEGs in *S. bambusicola* under 5-AC treatment at 0.8 mM. Unigenes were assigned to three categories: **(A)** biological processes, **(B)** cellular components, and **(C)** molecular functions.

In order to explore the transcriptional changes of *S. bambusicola* under 5-AC treatment, RNA-Seq analysis was conducted. A summary of sequencing and assembly data was presented in **Supplementary Table S2**. A total of 12,293 unigenes were assembled, with an average length of 2,513.92 bp and an *N_50_* of 3,662 bp. The length distribution of the unigenes was shown in **Supplementary Figure S4**. Functional annotation revealed that 86.11, 63.26, and 49.56% of the total unigenes were similar to known genes in the database of NR, Swiss-Prot and KOG, respectively (**Supplementary Table S3**). A total of 4,046 (32.91%) differentially expressed genes (DEGs) were identified, among which 2,392 unigenes (59.12%) were up-regulated and 1,654 unigenes (40.88%) were down-regulated under 5-AC condition (**Supplementary Tables S4**, **S5**). DEGs of 1,566 groups were categorized into three main independent classifications, which including “biological process,” “molecular function,” and “cellular component” (**Figure 3** and **Supplementary Table S6**). As shown in **Figure 3A** and **Supplementary Table S7**, for the biological process category, DEGs (66 unigenes) assigned to ‘pathogenesis’ (GO:0009405) was of the highest proportion, indicating 5-AC may influence the stress responses of *S. bambusicola*. Within the cellular component category (**Figure 3B** and **Supplementary Table S7**), most of DEGs were assigned to ‘integral component of membrane’ (GO:0016021) and ‘plasma membrane’ (GO:0005886), suggesting the changes in the composition and activity of plasma membrane induced by 5-AC. Within the molecular function category (**Figure 3C** and **Supplementary Table S7**), DEGs were mainly assigned into terms related to ‘oxidoreductase activity’ (GO:0016491 and GO:0016705), indicating the changes of ROS and redox-related enzymes involved in 5-AC-treated cultures.

### Effect of 5-AC on Expression of Genes Associated With Fluffy Mycelia Phenotype

**FIGURE 4:**
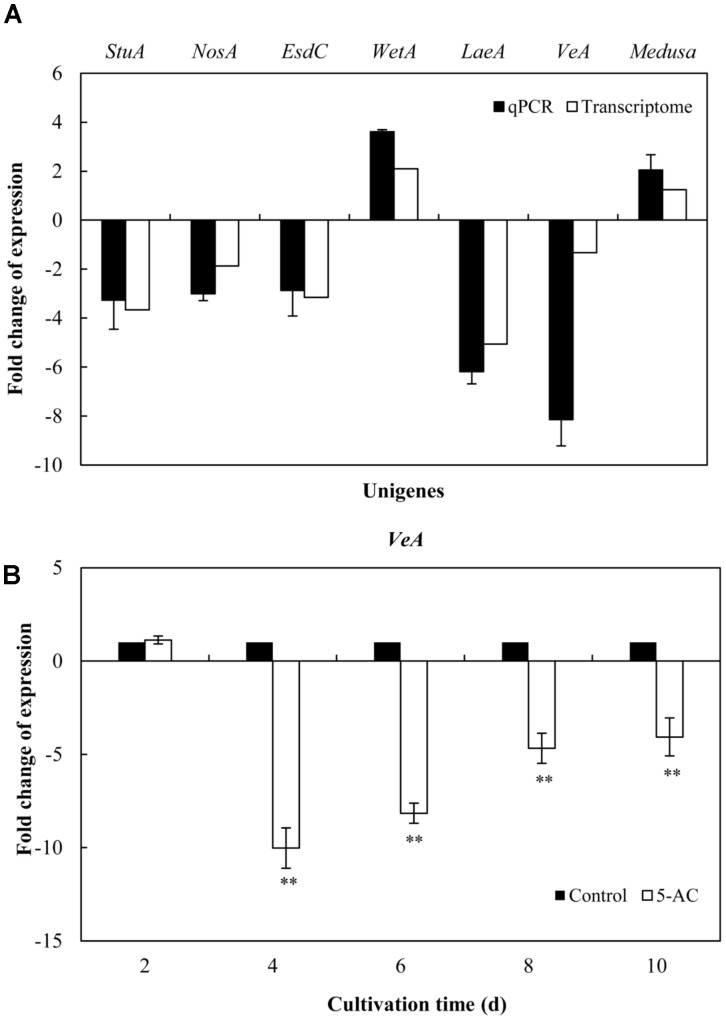
Effects of 5-AC on the expression of genes associated with fluffy mycelia phenotype of *S. bambusicola* S8. **(A)** Quantitative real-time PCR (qPCR) analysis of these genes expression in mycelia treated by 5-AC at 0.8 mM for 4 days. **(B)** Time profiles of expression of *VeA* gene in submerged cultures of *S. bambusicola* with or without addition of 5-AC. 5-AC was added on day 3 of the fermentation and cultured at 28°C. Values are mean ± SD from three independent experiments. ^∗∗^*p* < 0.01 versus control group.

On the basis of the results from previous studies (Wilkinson et al., 2011; Lin et al., 2013b), we investigated on the expression changes of unigenes associated with fluffy mycelia phenotype in our transcriptome data of *S. bambusicola* S8 (**Table 1**), including developmental regulator *FlbA* (CL105Contig2), *Apses* transcription factors (CL1361Contig1, CL1655Contig1, CL1915Contig1, comp4435_c0_seq1_3, and comp5120_c0_seq1_3), sexual development transcription factor *NosA* (CL1894Contig1), GTP-binding *EsdC* (CL3610Contig1), developmental regulatory protein *WetA* (CL453Contig1 and CL744Contig2), regulator *Medusa* (Comp1521_c0_seq1_3) and velvet complex subunit *VeA/LaeA* (CL5806Contig1, CL5736Contig1, and Comp426_c1_seq1_2). The treatment of 5-AC induced up- or down-regulation of the expression of *Apses* transcription factors and velvet complex subunit *LaeA*, while the developmental regulators such as *FlbA*, *WetA* and *Medusa* were up-regulated with the down-regulation of sexual development unigenes (activator *VeA and* transcription factor *NosA*). The expression levels of selected unigenes (comp4435_c0_seq1_3, CL1894Contig1, CL3610Contig1, CL744Contig2, Comp426_c1_seq1_2, CL5806Contig1, and Comp1521_c0_seq1_3) were validated using qRT-PCR (**Figure 4A**), which exhibited a consistent trend between the qRT-PCR and the transcriptome analyses. Based on the results of qRT-PCR validation, we found that the *VeA* gene was most significantly down-regulated (8.14-fold) by 5-AC (**Figure 4A**). During all cultural period, the most significant change of *VeA* expression induced by 5-AC occurred on day 4 (**Figure 4B**).

**Table 1:** The selected DEGs involved in the growth and development of *Shiraia bambusicola* under 5-AC treatment.

Unigene ID	Up/down	Fold change^a^	Description
Comp5120_c0_seq1_3	Up	2.46	Apses transcription factor-like protein [OAL03202.1]
CL1915Contig1	Up	4.35	Apses transcription factor [KNG50735.1]
CL105Contig2	Up	Inf	Developmental regulator FlbA [P38093.1]
CL744Contig2	Up	1.24	Developmental regulatory protein WetA [Q4WQL4.1]
CL453Contig1	Up	2.10	Regulatory protein WetA [ENOG4112AMK]
Comp1521_c0_seq1_3	Up	1.25	Regulator Medusa [ENOG4110G4V]
CL5736Contig1	Up	4.01	Velvet complex subunit LaeA [A2SUH3.1]
CL1655Contig1	Down	2.43	Apses transcription factor [ENOG4111JJU]
Comp4435_c0_seq1_3	Down	3.66	Apses transcription factor StuA [Q0U086.2]
CL1361Contig1	Down	2.00	Apses transcription factor-like protein [OAL47398.1]
CL1894Contig1	Down	1.87	C6 sexual development transcription factor-like protein NosA [OAL06581.1]
CL3610Contig1	Down	3.15	GTP-binding protein EsdC [ENOG4111JAY]
CL5806Contig1	Down	1.32	Sexual development activator VeA [ENOG4111FVS]
Comp426_c1_seq1_2	Down	5.06	Velvet complex subunit LaeA [A2SUH3.1]


*^a^Fold change, up: ratio(S1/S2); down: ratio(S2/S1). S1, the FPKM value of a unigene under 5-AC treatment; S2, the FPKM value of a unigene in control group. ‘Inf’ represents the unigene only expresses in 5-AC treatment groups.*

### Effect of 5-AC on Expression of Genes Associated With Hypocrellin Biosynthesis

**FIGURE 5:**
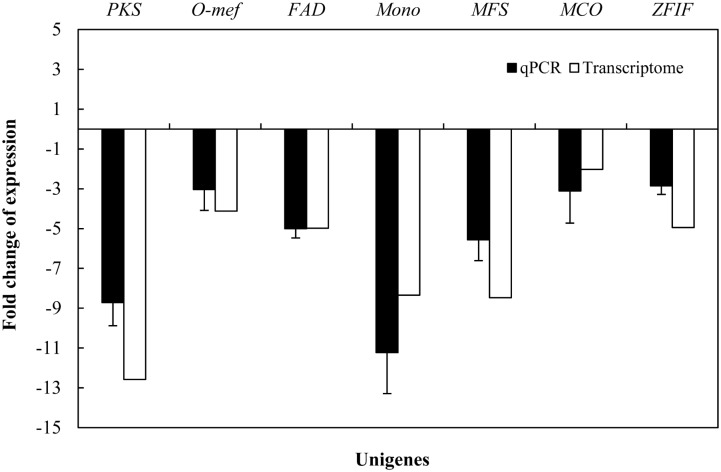
Effects of 5-AC on the expression of hypocrellin biosynthetic genes of *S. bambusicola* S8. 5-AC was added on day 3 of the culture. After 1 day of treatment the mycelia were harvested. Values are mean ± SD from three independent experiments.

Due to hypocrellin contents in mycelia were inhibited significantly by 5-AC (**Figure 1D**), putative 289 DEGs encoding enzymes involved in hypocrellin biosynthetic pathway were summarized in **Supplementary Table S8**. We found the unigene *PKS* (CL954Contig1), *Omef* (CL6443Contig1), *FAD* (CL2000Contig1), *Mono* (CL1046Contig1), and *MFS* (CL13Contig3 and CL5005Contig1) were annotated as polyketide synthase, *O*-methyltransferase, FAD/FMN-dependent oxidoreductase, monooxygenase and major facilitator superfamily, respectively with the highest similarity to the corresponding genes in the genome of *Shiraia* sp. Slf14 (AIW00658.1) (Yang et al., 2014), all of which were down-regulated clearly by 5-AC (**Table 2**). Furthermore, the expression levels of selected unigenes including *PKS* (CL954Contig1), *Omef* (CL6443Contig1), *FAD* (CL2000Contig1), *Mono* (CL1046Contig1), *MFS* (CL13Contig3), *MCO* (CL4891Contig1), and *ZFIF* (CL6402Contig1) were validated using qRT-PCR (**Figure 5**). After 5-AC treatment, the relative expression levels of the selected unigenes were down-regulated, among which *PKS* and *Mono* were more significantly down-regulated by 8.72- and 11.23-fold, respectively.

**Table 2:** Examples of differentially expressed unigenes involved in hypocrellin biosynthesis of *S. bambusicola* under 5-AC treatment.

Unigene ID	Up/down	Fold change^a^	Description
**Polyketide synthase (*PKS)***			
CL939Contig2	Down	4.10	Conidial yellow pigment biosynthesis polyketide synthase [XP_001939987.1]
CL954Contig1	Down	12.58	*Shiraia* sp. slf14 polyketide synthase [AIW00658.1]
***O*-Methyltransferase (*Omef)***			
CL6443Contig1	Down	4.12	*Shiraia* sp. slf14 *O*-methyltransferase [AIW00661.1]
CL6992Contig1	Down	4.77	*O*-Methyltransferase gsfB [D7PI16.1]
**FAD/FMA-dependent oxidoreductase (*FAD)***			
CL2000Contig1	Down	4.98	*Shiraia* sp. slf14 FAD/FMA-dependent oxidoreductase [AIW00665.1]
CL237Contig1	Down	7.32	Predicted FAD-dependent oxidoreductase [KOG2665]
**Monooxygenase (*Mono)***			
CL1046Contig1	Down	8.34	*Shiraia* sp. slf14 hydroxylase [AIW00664.1]
CL1294Contig1	Down	2.23	Cytochrome P450 monooxygenase aclC [Q2UPB1.1]
**Major facilitator superfamily (*MFS)***			
CL13Contig3	Down	8.47	*Shiraia* sp. slf14 major facilitator superfamily (MFS) transporter [AIW00660.1]
CL5005Contig1	Down	4.77	*Shiraia* sp. slf14 major facilitator superfamily (MFS) transporter [AIW00668.1]
**Multicopper oxidase (*MCO)***			
CL3495Contig1	Down	9.14	Multicopper oxidase aurL2 [I1RF64.1]
CL4891Contig1	Down	2.03	Multicopper oxidase [XP_007708085.1]
**Zinc finger transcription factor (*ZFIF)***			
CL6402Contig1	Down	4.95	Zinc finger transcription factor 37 [Q5A4F3.1]


*^a^Fold change: ratio(S2/S1). S1, the FPKM value of a unigene under 5-AC treatment; S2, the FPKM value of a unigene in control group.*

.

### Effects on ROS Generation, Activities of Redox-Related Enzymes, and Gene Expression

**FIGURE 6:**
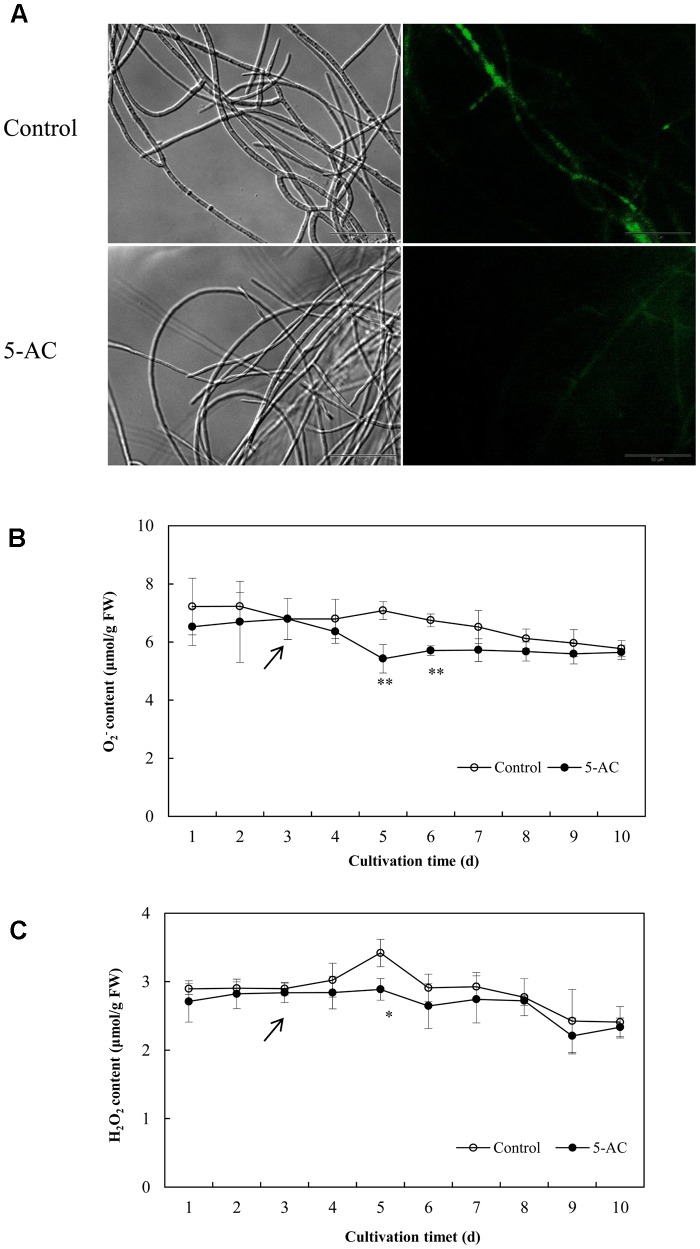
Effects of 5-AC on ROS generation of *S. bambusicola* S8. **(A)** Bright-field image (left) and fluorescence microscopy (485 nm excitation and 528 nm emission) of DCFH-DA-stained mycelium (right) in cultures with or without 5-AC. 5-AC at 0.8 mM was added on day 3 of the culture. After 2 days of treatment the mycelia were harvested and observed. The O_2_^-^ **(B)** and H_2_O_2_ **(C)** contents of mycelia were detected under 5-AC treatment. *Arrow* indicates the time point of 5-AC addition. Values are mean ± SD from three independent experiments. ^∗^*p* < 0.05, ^∗∗^*p* < 0.01 versus control.

**FIGURE 7:**
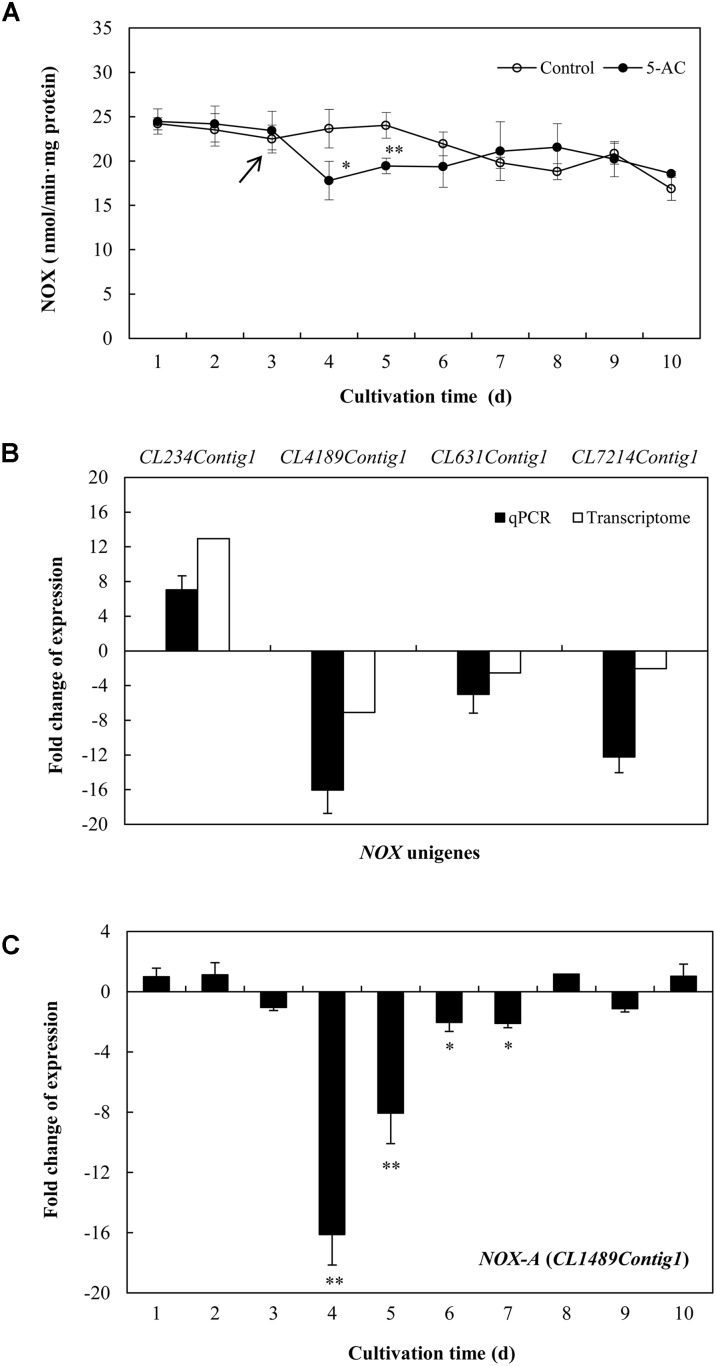
**(A)** Effects of 5-AC on the activity of NADPH oxidase (NOX) of *S. bambusicola* S8. 5-AC at 0.8 mM was added on day 3 of the culture. *Arrow* indicates the time point of 5-AC addition. **(B)** Effects of 5-AC on the expression of different *NOX*-related genes. **(C)** Time profiles of expression of *NOX-A* gene (CL4189Contig1) in submerged cultures of *S. bambusicola* with or without addition of 5-AC. 5-AC at 0.8 mM was added on day 3. After 1 day of treatment the mycelia were harvested and measured. Values are mean ± SD from three independent experiments. ^∗^*p* < 0.05, ^∗∗^*p* < 0.01 versus control.

The ROS generation in mycelia was observed using fluorescence microscopy directly. After 5-AC treatment, the green fluorescence was weakened in DCFH-DA-stained cells (**Figure 6A**), indicating the attenuation of ROS production. The content of O_2_^-^ significantly dropped down to the lower level of 5.42 μmol/g FW on day 5, which was 23.45% lower than that of control (**Figure 6B**). The trend of H_2_O_2_ production was similar to the change of O_2_^-^ content in mycelia (**Figure 6C**). H_2_O_2_ production decreased remarkably by 15.50% after 2-day treatment of 5-AC. Moreover, we found that the activity of NOX decreased significantly within 2 days of the treatment (**Figure 7A**). However, there were no significant changes of the activities of CAT, SOD, and GSH-Px (**Supplementary Figure S5**). According to the *de novo* sequencing and comparative analysis, there were 22 DEGs significantly annotated in oxidoreductase activity (**Table 3** and **Supplementary Table S9**). Among them, four unigenes (CL234Contig1, CL4189Contig1, CL631Contig1, and CL7214Contig1) were classified to the NADPH oxidase (*NOX*), of which three unigenes were down-regulated by 5-AC (**Figure 7B**). *NOX-A* gene (CL4189Contig1) was most down-regulated (16.04-fold). During all cultural period, the most significant depression on *NOX-A* expression occurred on day 4 (**Figure 7C**).

**Table 3:** Differentially expressed unigenes involved in ROS biosynthesis and antioxidant enzyme activity of *S. bambusicola* under 5-AC treatment.

Unigene ID	Up/down	Fold change^a^	Description
**NADPH oxidase (*NOX)***			
CL234Contig1	Up	12.94	Ferric reductase, NADH/NADPH oxidase and related proteins [KOG0039]
CL4189Contig1	Down	7.11	NADPH oxidase A, NOX-A[OAK97727.1]
CL631Contig1	Down	2.54	Ferric reductase, NADH/NADPH oxidase and related proteins [KOG0039]
CL7214Contig1	Down	2.04	Ferric reductase, NADH/NADPH oxidase and related proteins [KOG0039]
**Catalase (*CAT)***			
CL2865Contig2	Up	2.46	Catalase catB [PTT_14006]
CL7542Contig1	Up	2.18	Catalase/peroxidase HPI [OAK97221.1]
**Superoxide dismutase (*SOD)***			
CL5247Contig1	Up	2.15	Copper/zinc binding superoxide dismutase[OAG19079.1]
**Glutathione peroxidase (*GSH-Px)***			
CL3250Contig1	Up	7.69	Glutathione peroxidase activity [GO:0004602]
CL6027Contig1	Up	11.26	Glutathione peroxidase activity [GO:0004602]
**Thioredoxin peroxidase (*TPx)***			
CL3922Contig1	Up	5.16	Thioredoxin peroxidase [Q9Y7F0.1]
**Peroxidase**			
CL1823Contig1	Up	3.68	Peroxidase/oxygenase [KOG2408]
CL1823Contig2	Up	12.68	Peroxidase/oxygenase [KOG2408]
CL2875Contig1	Down	3.94	Peroxidase/oxygenase [KOG2408]
CL2990Contig1	Down	117.61	Peroxidase/oxygenase [KOG2408]
CL400Contig2	Down	8.98	Peroxidase/oxygenase [KOG2408]
CL4223Contig1	Down	2.70	Peroxidase [KZM21430.1]
CL485Contig1	Down	6.86	Versatile peroxidase VPL1 [Q9UR19.1]
CL6073Contig1	Up	3.58	Cytochrome c peroxidase [Q7SDV9.1]
CL7536Contig1	Down	4.45	Peroxidase/oxygenase [KOG2408]
CL8042Contig1	Down	24.28	Manganese peroxidase mpn [SNOG_01153]
CL9247Contig1	Up	7.76	Cloroperoxidase [OAG20559.1]
comp928_c0_seq1_2	Down	2.28	Peroxidase/oxygenase [KOG2408]


*^*a*^Fold change, up: ratio(S1/S2); down: ratio(S2/S1). S1, the FPKM value of a unigene under 5-AC treatment; S2, the FPKM value of a unigene in control group.*

### The Role of ROS in Reduced Hypocrellin Production by 5-AC

**FIGURE 8:**
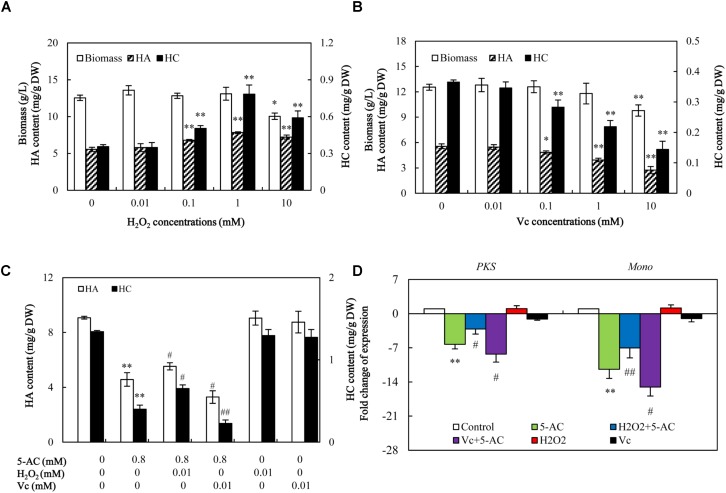
The effect of decreased ROS on the mycelial growth, hypocrellin production, and related gene expression after 5-AC treatment. Effects of H_2_O_2_ **(A)** and Vc **(B)** on mycelial biomass and hypocrellin contents. H_2_O_2_ and Vc were added on day 3 of the culture. After 5 days of treatment the mycelia were harvested. Effects of H_2_O_2_ and Vc on hypocrellin contents **(C)** and gene expression **(D)** after 5-AC treatment. H_2_O_2_ (0.01 mM) and Vc (0.01 mM) were added 1 h prior to 5-AC treatment at 0.8 mM on day 3. Hypocrellin contents and the gene expression levels were measured on day 8 and day 4, respectively. *PKS*: polyketide synthase (CL954Contig1), *Mono*: monooxygenase (CL1046Contig1). Values are mean ± SD from three independent experiments. ^∗^*p* < 0.05, ^∗∗^*p* < 0.01 versus control, ^#^*p* < 0.05, ^##^*p* < 0.01 versus 5-AC alone group.

We used ROS donor (H_2_O_2_) and scavenger vitamin C (Vc) to investigate the role of ROS in 5-AC-induced reduction of hypocrellin production. As shown in **Figure 8A**, H_2_O_2_ treatment increased the hypocrellin contents in a dose-dependent manner (0–1 mM), but inhibited the fungal growth at 10 mM. Contrarily, Vc treatment exhibited significant inhibition on hypocrellin production at 0.1–10 mM and the mycelial growth at 10 mM (**Figure 8B**). To avoid the disturbance by its direct action of H_2_O_2_ or Vc on fungal growth and metabolite, we chose 0.01 mM of H_2_O_2_ or Vc for ROS donor or scavenger. At this content, H_2_O_2_ or Vc could increase or decrease ROS production (**Supplementary Figure S6**), but it alone exerted no influence on the fungal growth and metabolites (**Figures 8A,B**). When S8 stain were cultured in the presence of H_2_O_2_ at 0.01 mM for 1 h prior to 5-AC addition, significant rescues of the expression levels of key enzyme genes (*PKS* and *Mono*), and hypocrellin contents (HA from 4.57 to 5.53 mg/g DW and HC from 0.40 to 0.65 mg/g DW) were observed on day 8 (**Figures 8C,D**). After pre-incubation with Vc at 0.01 mM for 1 h prior to 5-AC addition, the contents of HA and HC in mycelia showed a much more serious decrease (**Figure 8C**). Simultaneously, the expression levels of *PKS* and *Mono* were further down-regulated by 32.01 and 31.87%, respectively (**Figure 8D**). This set of results is indicative of the relationship between ROS and 5-AC-induced depression of hypocrellin in *S. bambusicola*.

## Discussion

DNA methylation is one of the most crucial epigenetic modifications to regulate fungal growth and biosynthesis of secondary metabolites (Martienssen and Vincent, 2001). Through a chemical epigenetic approach with the DNA methyltransferase inhibitor 5-AC, the epigenetic regulatory mechanism on biosynthetic genes and structurally unique secondary metabolites has been discovered recently. Huang et al. (2017) have demonstrated that inhibition of DNA methyltransferase activity by 5-AC led to growth inhibition of *Pleurotus ostreatus* dikaryons with less radial extension and a smaller mycelial colony, but activation of the accumulation of yellow pigments. 5-AC was reported to inhibit protoperithecia development, stimulate conidia formation, and enhance carotenoid production of the filamentous fungus *N. crassa* (Kritskii et al., 2001). Addition of 5-AC triggered a biosynthesis of two new polyketides in *Diatrype* sp., and also elicited the production of several oxylipins of *Cladosporium cladosporioides*, suggesting a positive modulation on secondary metabolite biosynthesis (Williams et al., 2008). On the other hand, the effects of 5-AC on fungi lacking DNA methylation have also been reported. 5-AC induced a fluffy phenotype and the loss of aflatoxin production in *Aspergillus* species lacking DNA methylation or at extremely low level (Wilkinson et al., 2011; Lin et al., 2013a). In present study, both HA and HC contents in *S. bambusicola* mycelium were inhibited by 5-AC (**Figure 1C**). Both the RNA-seq data and qRT-PCR analysis demonstrated that 5-AC down-regulated the expression level of some key genes including *PKS* (CL954Contig1), *Omef* (CL6443Contig1), *FAD* (CL2000Contig1), *Mono* (CL1046Contig1), *MFS* (CL13Contig3), *MCO* (CL4891Contig1), and *ZFIF* (CL6402Contig1) in the later steps of hypocrellin biosynthesis (**Figure 5**, **Table 2**, and **Supplementary Table S8**). These genes encode enzymes to catalyze condensation reaction of acetyl-CoA and malonyl-CoA subunits, the polyketide oxidation and modification of methyl groups in the biosynthesis of hypocrellin (Zhao et al., 2016). However, both the electrophoretogram of DNA McrBC digestion (**Figure 2A**) and chromatography of 5-mdC (**Figure 2B**) demonstrated that the DNA methylation is absent in *S. bambusicola* mycelium. Hence, it is unlikely that the 5-AC plays a role as a DNA methylation inhibitor in *S. bambusicola* mycelium cultures.

In our study, 5-AC treatment induced the stain to the white “fluffy” phenotype (**Figure 1A**), which was consistent with those found in *Aspergillus* species in response to 5-AC treatment (Ben-Ami et al., 2010). The transcriptional analysis on *Aspergillus* “fluffy” phenotype revealed the expression changes of multiple genes involved in coordinating fungal development and secondary metabolism (Wilkinson et al., 2011; Lin et al., 2013b). In our results from both the RNA-seq data and qRT-PCR analysis, transcription levels of the selected unigenes involved in conidial development, such as *StuA* and *EsdC* decreased to a certain extent (**Figure 4A** and **Table 1**), which was consistent with those found in *A*. *flavus* in response to 5-AC treatment (Lin et al., 2013b). Two genes (*VeA* and *NosA*) encoding sexual development proteins were down-regulated significantly (**Figure 4A** and **Table 1**). In addition, it was worth noting that *VeA* gene, encoding sexual developmental and secondary metabolism regulator, was most down-regulated (8.14-fold) significantly by 5-AC (qPCR in **Figure 4A**). Simultaneously, the expression level of *LaeA* gene of *S. bambusicola* were down-regulated significantly by 5-AC (**Figure 4A**). To our best knowledge, *VeA* could bridge the *VelB* and *LaeA* to form the VelB-VeA-LaeA (velvet) complex to co-regulate fungal development and secondary metabolism (Bayram et al., 2008). In our research, the most significant change of *VeA* expression induced by 5-AC occurred on day 4 (**Figure 4B**). These findings suggesting that 5-AC could induce fluffy phenotype of *S. bambusicola* via regulating velvet complex. We supposed that the suppression of *VeA* and *LaeA* genes may make an insufficient amount of VeA and LaeA protein to form the velvet complex, leading to a block of conidium formation and some secondary metabolites production. In conclusion, 5-AC could lead to dysregulation of the expression pattern of genes involved in asexual sporulation and growth of vegetative hyphae.

5-AC has been used as an antineoplastic drug due to its cytotoxic effects and DNA hypomethylation (Kaminskas et al., 2005). ROS production was found to be a marker for cytotoxicity of 5-AC in leukemic cells (Gao et al., 2008). However, a detectable attenuation in the production of ROS was found in *A. flavus* after 5-AC treatment (Yang et al., 2015). In present study, the content of O_2_^-^ and H_2_O_2_ was decreased significantly (**Figure 6**). It was reported that NADPH oxidase, a trans-membrane protein, could transfer electrons from NADPH to oxygen molecular and generate ROS (Scott and Eaton, 2008). We also found 5-AC treatment down-regulated the expression level of *NOX* gene and inhibited NOX activity in *S. bambusicola* (**Figure 7**). NOX-A gene (CL4189Contig1) was down-regulated by 16.04-fold induced by 5-AC (qPCR in **Figure 7B**). Furthermore, there were no significant changes of the activities of CAT, SOD, and GSH-Px (**Supplementary Figure S5**). Hence, we propose that ROS reduction induced by 5-AC was mainly due to the inhibition on *NOX*. On the other hand, Zhang et al. (2014) reported that hypocrellin production was increased from 110.0 to 408.5 mg/L after the additional of 30 μM H_2_O_2_. Deng et al. (2016) found that the addition of high H_2_O_2_ concentrations (10 and 20 mM) could also increase hypocrellin production by 27 and 25%, respectively after 72 h incubation (Deng et al., 2016). With ROS donor H_2_O_2_ and scavenger Vc, we demonstrated ROS was involved in the inhibition role of 5-AC on biosynthetic gene expression and hypocrellin accumulation (**Figure 8C**). In addition to its constitutive role as a DNA methyltransferase inhibitor, 5-AC was proposed to regulate histone (Komashko and Farnham, 2010), induce DNA mutation (Doiron et al., 1999) and remodel chromatin (West et al., 2008). Whether such events are associated with 5-AC acting on hypocrellin biosynthesis needs to be further investigated.

## Conclusion

To our knowledge, this is a first report on levels of DNA methylation of *S. bambusicola* mycelium. We found that *S. bambusicola* is a species lacking DNA methylation during vegetative growth. However, a DNA methylation inhibitor 5-AC still induced the fluffy phenotype and inhibited hypocrellin biosynthesis of this fungus. The fluffy phenotype in 5-AC-treated *S. bambusicola* may be due to the strong modulation of conidial/sexual developmental regulator genes such as *StuA* and *WetA* and the repression of the essential developmental regulator genes *VeA* and *LaeA* genes. It was a surprise finding that 5-AC reduced ROS production significantly in the mycelia via the inhibition on *NOX* gene expression and NOX activity. Our present work revealed the significant role of ROS in 5-AC induced decrease of hypocrellins in *S. bambusicola*. Our results provide a reference of ROS inhibition by 5-AC for future studies using 5-AC as a specific DNA methylation inhibitor on fungal growth and metabolite biosynthesis.

## Author Contributions

JW and YM were the recipients of funds and conceived the experiment. YM and CL undertook experiments and data analysis. JW and YM prepared the manuscript. All authors have read and approved the final manuscript.

## Conflict of Interest Statement

The authors declare that the research was conducted in the absence of any commercial or financial relationships that could be construed as a potential conflict of interest.

## Footnotes

1 ftp://ftp.ncbi.nih.gov/blast/db

2 http://www.uniprot.org/downloads

3 ftp://ftp.ncbi.nih.gov/pub/COG/KOG/kyva

4 http://www.genome.jp/kegg/

5 http://www.geneontology.org/

6 http://www.ncbi.nlm.nih.gov/geo/
