# Supplementary material for: Effects of 5-Azacytidine on Growth and Hypocrellin Production of Shiraia bambusicola
Source: Front Microbiol. 2018 Oct 18;9:2508. doi: 10.3389/fmicb.2018.02508 (PMC6200910; doi:10.3389/fmicb.2018.02508)
Supplement: Supplementary file 1 [file Data_Sheet_1.PDF]

Supplemental Information

Effects of 5-Azacytidine on Growth and Hypocrellin Production of *Shiraia bambusicola*

Yan Jun Ma, Can Song Lu, Jian Wen Wang
College of Pharmaceutical Sciences, Soochow University, Suzhou 215123, China

✉ Jian Wen Wang
jwwang@suda.edu.cn; bcjwwang@gmail.com

TABLE S1| The primers of the target genes and the internal reference gene used for qRT-PCR. F: forward primer, R: reverse primer.

**Unigene ID**	**Gene description**	**Sequence**
18S	Reference gene	F: 5’-ACGCAGCGAAATGCGATAAG-3’<br>R: 5’-CAAATTGTGCTGCGCTCCAA-3’
Comp426_c1_seq1_2	Velvet complex subunit LaeA	F: 5’-CTATTACGCGCCAGTAGCCA-3’<br>R: 5’-TATCCGGTTGATCTGCGACG-3’
Comp1521_c0_seq1_3	Regulator Medusa	F: 5’-GGCGTTTGGGTCATTGCTTT-3’<br>R: 5’-GGCAAATGAAGGCTGTGCAA-3’
Comp4435_c0_seq1_3	Apses transcription factor StuA	F: 5’-CCACCCGTCCTCTAGGTACA-3’<br>R: 5’-GAGGCAGGTGTTTTGCTGTG-3’
CL744Contig2	Developmental regulatory protein WetA	F: 5’-CAAAAGCGCACAGTCGGAAA-3’<br>R: 5’-CCTGCCTTTTTGTGTCGTGG-3’
CL1894Contig1	C6 sexual development transcription factor-like protein NosA	F: 5’-CAACAACGAGCAAAGGCGAA-3’<br>R: 5’-CCGTGGTTGGTGTAGTCGAA-3’
CL3610Contig1	GTP-binding protein EsdC	F: 5’-CAAAATGGCCGCTATCCAGC-3’<br>R: 5’-TAGCAACGGAGGCAGTTGAG-3’
CL5806Contig1	Sexual development activator VeA	F: 5’-ATACGCCCAGTCTCCACTCT-3’<br>R: 5’-AAACCTTGAGCAGCAAAGCG-3’
CL13Contig3	*Shiraia* sp. slf14 major facilitator superfamily (MFS) transporter	F: 5’-TCCATTGTTCCAGGCGTACC-3’<br>R: 5’-TATGCCTCGCCGTTCTTTGT-3’
CL954Contig1	*Shiraia* sp. slf14 polyketide synthase	F: 5’-GCTGTCCTGAAACGACTGGA-3’<br>R: 5’-CGAATATCGGGCACGTCTGA-3’
CL1046Contig1	*Shiraia* sp. slf14 hydroxylase	F: 5’-GGACGATTCCACGCGATTTG-3’<br>R: 5’-CATGGCTGACCGCATCATTG-3’
CL2000Contig1	*Shiraia* sp. slf14 FAD/FMA-dependent oxidoreductase	F: 5’-GATGGTTGCGTTGGCAAGTT-3’<br>R: 5’-GCTTCCCACCCATACGACAA-3’
CL4891Contig1	Multicopper oxidase	F: 5’-CCCATCAAACCATTCGTCGC-3’<br>R: 5’-ATTTTGTGGCCGAGGTCCAT-3’
CL6402Contig1	Zinc finger transcription factor 37	F: 5’-TACGATGAGCGCCAACAACT-3’<br>R: 5’-CTGCATGGCTATCGGGGATT-3’
CL6443Contig1	*Shiraia* sp. slf14 *O*-methyltransferase	F: 5’-GCTGGTGGACCTCTCCTTTC-3’<br>R: 5’-AGCGTGCCTTCAGGTAGTTC-3’
CL234Contig1	Ferric reductase, NADH/NADPH oxidase and related proteins	F: 5’-GAAGCGCTGACTGTGCAAAA-3’<br>R: 5’-GATGGATCTTGCGTTTCGCC-3’
CL631Contig1	NADPH oxidase A, NOX-A	F: 5’-CTGCTATACCGCCCTCACTG-3’<br>R: 5’-AGCGAACGAAGAAGAGCACA-3’
CL4189Contig1	Ferric reductase, NADH/NADPH oxidase and related proteins	F: 5’-CAGGTACTCGGATCACGCTC-3’<br>R: 5’-GCCGTCAGTACCCAGTATCG-3’
CL7214Contig1	Ferric reductase, NADH/NADPH oxidase and related proteins	F: 5’-CATGCACCAGAGTCATCCGT-3’<br>R: 5’-CTCACGAACTGCCCATACGA-3’

TABLE S2| Illumina RNA-Seq reads and *de novo* assembly statics of *S. bambusicola*.

Attributes	Control			5-AC		
	Sample-1	Sample-2	Sample-3	Sample-1	Sample-2	Sample-3
**Total clean reads**	44955902	48342102	46551774	45032460	48475264	49017906
**Total mapped reads**	44570791 (99.14%)	47861314 (99.01%)	46130507 (99.10%)	44637772 (99.12%)	47974891 (98.97%)	48519993 (98.98%)
**Total unigenes (≥300 bp)**	12293					
**Max length**	22967					
**Min length**	301					
**Average length**	2513.92					
**N50**	3662					

TABLE S3| Summary statistics of unigenes annotation numbers.

Annotation	Database				
	NR	Swiss-Prot	KOG	KEGG	GO
Numbers	10585	7776	6092	4099	7601

TABLE S4| Summary of DEGs in 5-AC treatment mycelium of *S. bambusicola*. The up/down-regulated DEGs were detected by comparing RNA-seq data of the unigenes of 5-AC treatment group to control group (up: ratio>1), down: ratio<1).

Control	Case	Up-regulated	Down-regulated	Total DEGs
Untreated group	5-AC-treated group	2392	1654	4046

Figure S1

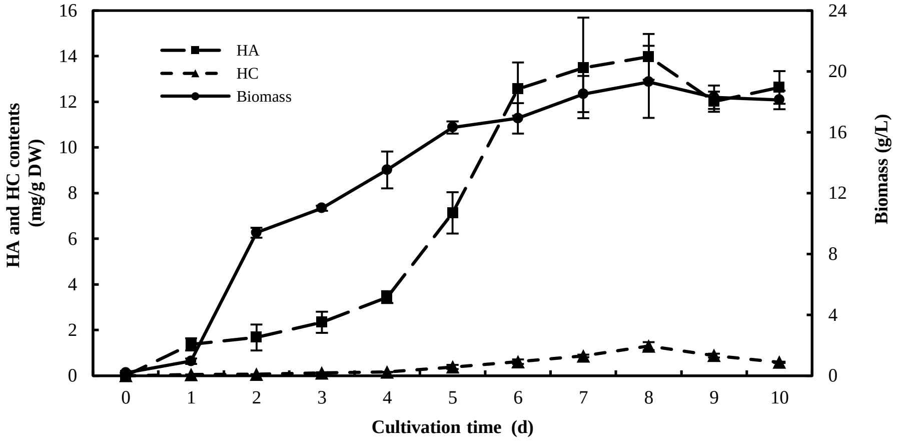

FIGURE S1| Time profiles of mycelia biomass and hypocrellin contents in submerged cultures of *S. bambusicola* S8. Values are mean ± SD from three independent experiments.

Figure S2

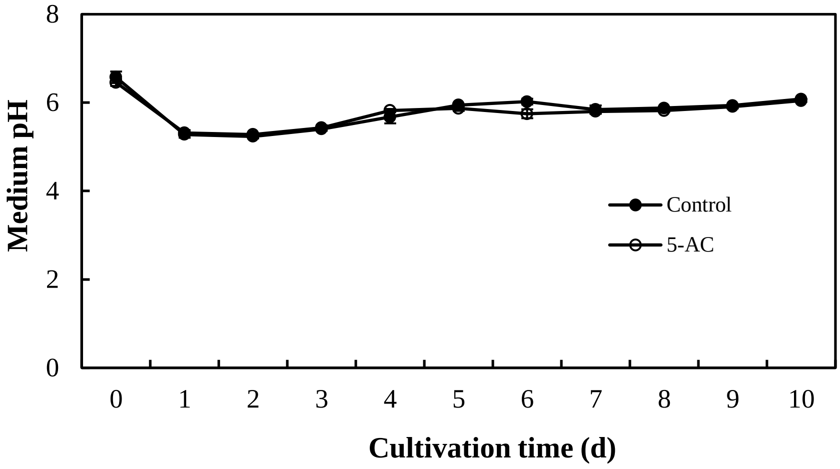

FIGURE S2| Effect of 5-AC on medium pH of *S. bambusicola* S8. 5-AC was added on day 3 of the culture. Values are mean ± SD from three independent experiments.

Figure S3

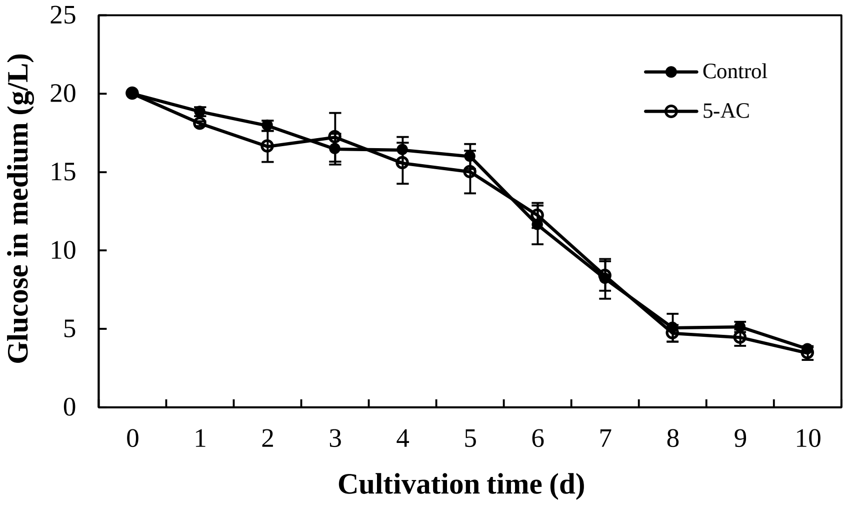

FIGURE S3| Effect of 5-AC on glucose consumption of *S. bambusicola* S8. 5-AC was added on day 3 of the culture. Values are mean ± SD from three independent experiments.

Figure S4

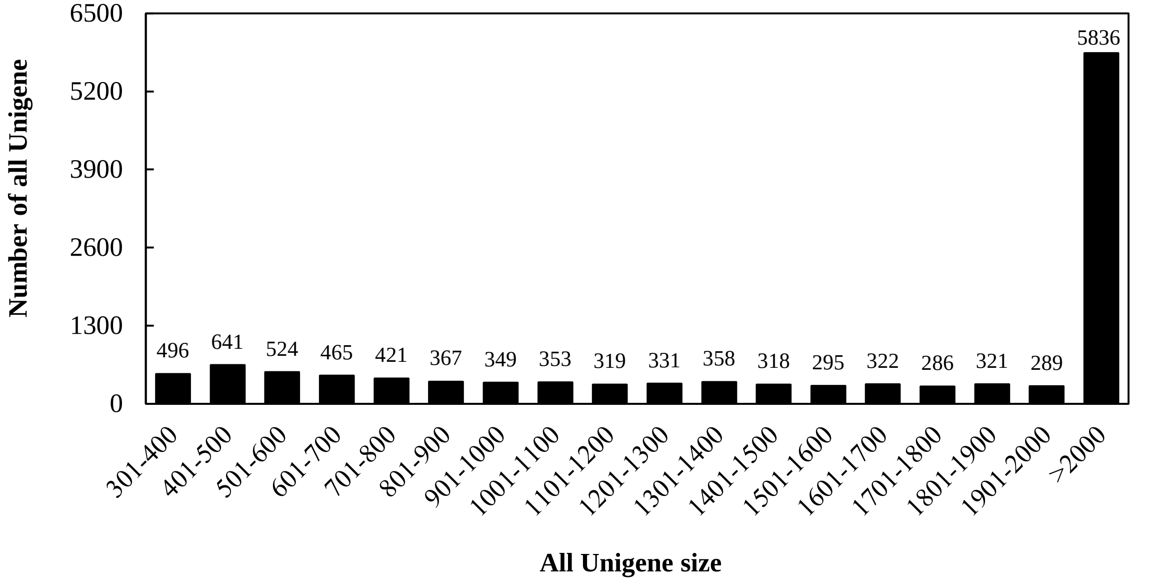

FIGURE S4| Length distributions of the unigenes.

Figure S5

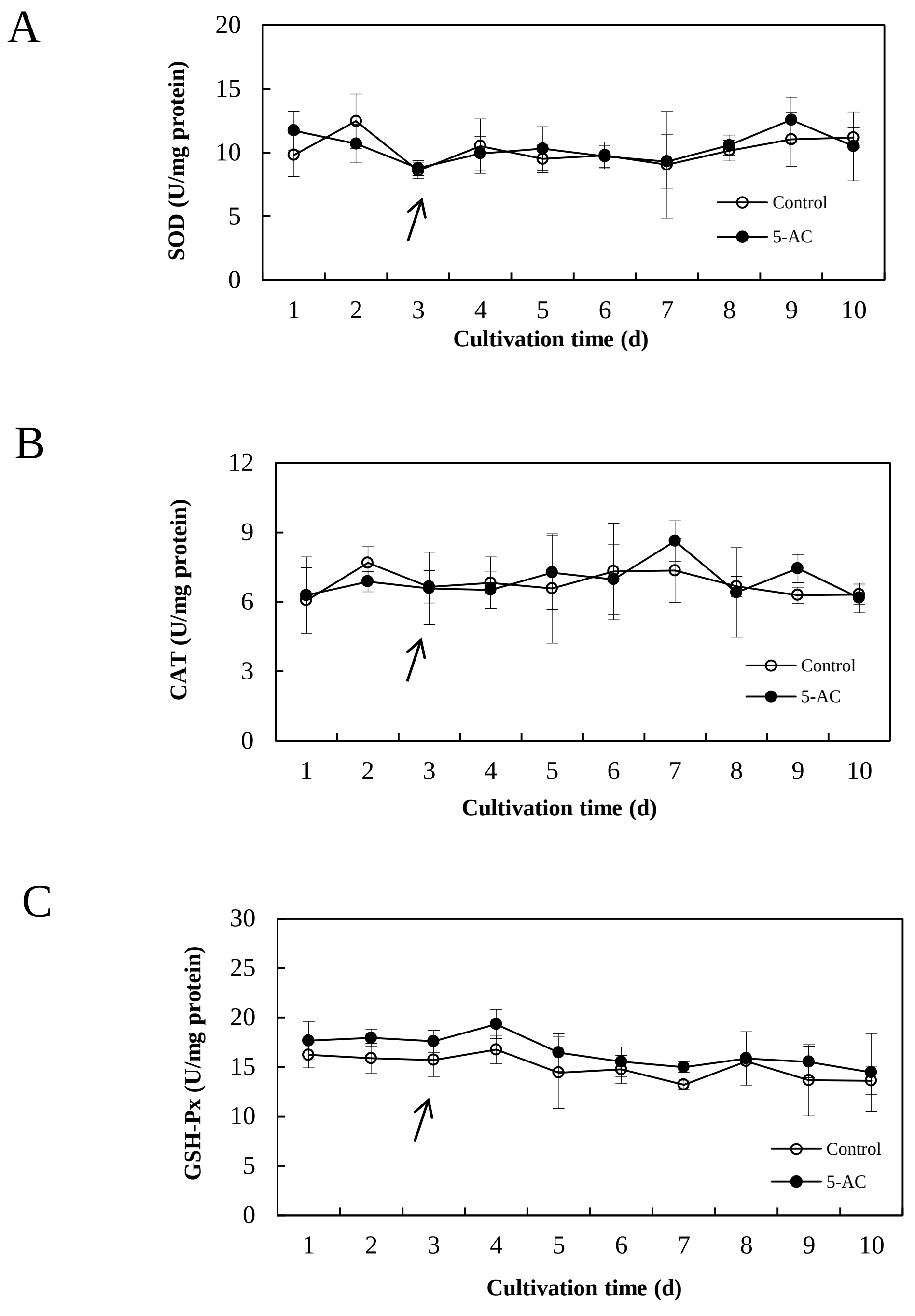

FIGURE S5| Effects of 5-AC on antioxidant enzyme activities of *S. bambusicola* S8. The superoxide dismutase (SOD) **(A)**, catalase (CAT) **(B)**, and glutathione peroxidase (GSH-Px) **(C)** activities of mycelia were detected under 5-AC treatment. 5-AC (0.8 mM) was added on day 3 of the culture. *Arrow* indicates the time point of 5-AC addition. Values are mean ± SD from three independent experiments.

Figure S6

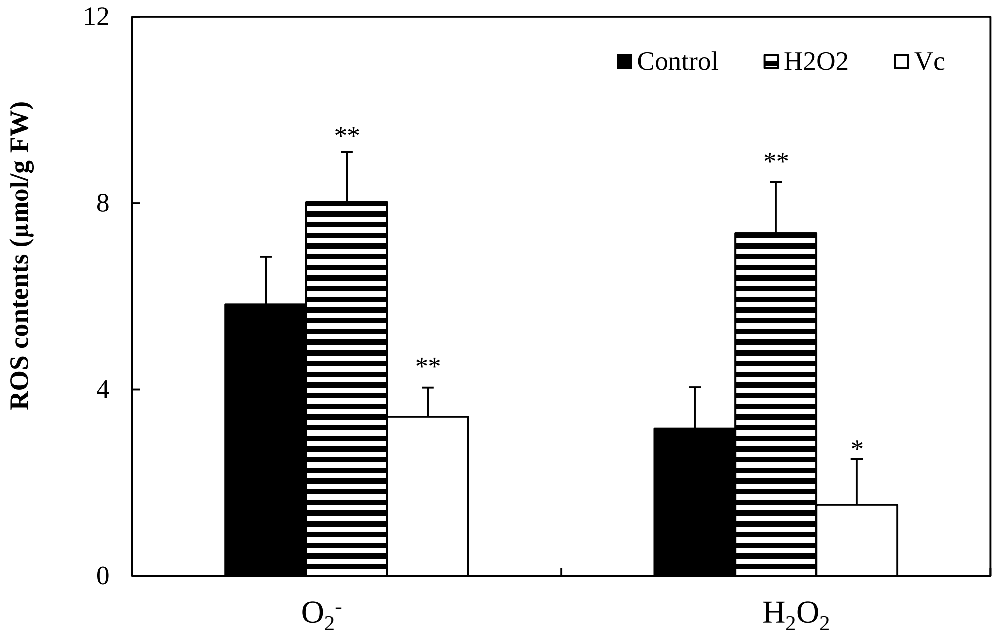

FIGURE S6| Effects of exogenous H_2O_2 and Vc at 0.01 mM on ROS (O_2^- and H_2O_2) contents in *S. bambusicola* S8 mycelium. H_2O_2 or Vc was added on day 3 of the culture. After 1 day of treatment the mycelia were harvested and measured. Values are mean ± SD from three independent experiments. *p < 0.05, **p < 0.01 versus control.
